# Supplementary material for: Simultaneous confidence intervals for an extended Koch-Röhmel design in three-arm non-inferiority trials
Source: Stat Methods Med Res. 2023 Jul 28;32(9):1784–98. doi: 10.1177/09622802231189592 (PMC10540495; doi:10.1177/09622802231189592)
Supplement: sj-pdf-1-smm-10.1177_09622802231189592 - Supplemental material for Simultaneous confidence intervals for an extended Koch-Röhmel design in three-arm non-inferiority trials [file sj-pdf-1-smm-10.1177_09622802231189592.pdf]

Table 1. Simulation results for probability of success (PoS) in different scenarios, nominal $\alpha = 2.5\%$, $\sigma = 2$, $\mu_P = 0$, historical reference mean $\mu_R^{hist} = 1$, $\delta_0 = 0.5$, number of simulation replicas $100.000.000$, Sample size: $N = n_E + n_R + n_P = 356 + 348 + 145 = 849$. First 5 rows correspond to scenarios in which H_{EP}^S or H_{ER}^N (or both) are true (PoS = type I error). In the last two rows, H_{EP}^S and H_{ER}^N are false.

		Filter satisfied		Probability of Success			
μ_E	μ_R	IU	superiority	Without SCI	IU	Informative	Single-Step
-0.5	-1	0.000	0.000	0.000	0.000	0.000	0.000
0	0.25	0.042	0.243	0.016	0.005	0.006	0.007
0	0.75	0.964	0.537	0.000	0.000	0.000	0.000
0	0.5	0.323	0.715	0.003	0.002	0.001	0.001
0.25	0.75	0.790	0.967	0.015	0.011	0.007	0.006
0.75	1.25	1.000	1.000	0.025	0.025	0.024	0.013
0.25	0.5	0.323	0.715	0.134	0.071	0.071	0.080
0.75	1	0.981	0.999	0.378	0.367	0.357	0.277

Table 2. Simulation results for coverage probability of SCIs in different scenarios, nominal $\alpha = 2.5\%$, $\sigma = 2$, $\mu_P = 0$, historical reference mean $\mu_R^{hist} = 1$, $\delta_0 = 0.5$, number of simulation replicas $100.000.000$, Sample size: $N = n_E + n_R + n_P = 356 + 348 + 145 = 849$, sim.=simultaneous coverage probability.

		IU			Informative			Single-step		
μ_E	μ_R	L_{ER}	L_{EP}	sim.	L_{ER}^{inf}	L_{EP}^{inf}	sim.	L_{ER}^S	L_{EP}^S	sim.
-0.5	-1	1.000	0.975	0.975	1.000	0.975	0.975	0.987	0.987	0.976
0	0.25	1.000	0.975	0.975	0.999	0.975	0.975	0.987	0.987	0.976
0	0.75	0.998	0.975	0.975	0.998	0.975	0.975	0.987	0.987	0.975
0	0.5	0.997	0.975	0.975	0.997	0.975	0.975	0.987	0.987	0.976
0.25	0.75	0.985	1.000	0.985	0.985	0.999	0.985	0.987	0.987	0.976
0.75	1.25	0.975	1.000	0.975	0.975	0.999	0.975	0.987	0.987	0.976
0.25	0.5	0.997	0.997	0.997	0.995	0.993	0.989	0.987	0.987	0.976
0.75	1	0.976	1.000	0.976	0.992	0.993	0.986	0.987	0.987	0.975
